# Evaluating *Trans*‐Benzocyclobutene‐Fused Cyclooctene as a Monomer for Chemically Recyclable Polymer

**DOI:** 10.1002/asia.202201133

**Published:** 2023-01-03

**Authors:** Hsin‐Wei Su, Junfeng Zhou, Seiyoung Yoon, Junpeng Wang

**Affiliations:** ^1^ School of Polymer Science and Polymer Engineering The University of Akron 170 University Ave Akron Ohio 44325 USA

**Keywords:** polymerization thermodynamics, fused-ring cyclooctenes, benzo-effect, chemically recyclable polymers, olefin metathesis

## Abstract

Chemically recyclable polymers offer a promising solution to address the issues associated with the unsustainable use of plastics by converting the traditional linear plastic economy into a circular one. Central to developing chemically recyclable polymers is to identify the appropriate monomers that enable practical conditions for polymerization and depolymerization and ensure useful stability and material properties. Our group has recently demonstrated that *trans*‐cyclobutane‐fused cyclooctene (*t*CBCO) meets the abovementioned requirements and is a promising candidate for developing chemically recyclable polymers. Herein, encouraged by the success with *t*CBCO, we investigate the thermodynamics of polymerization of a relevant system, *trans*‐benzocyclobutene‐fused‐cyclooctene, which can be viewed as *t*CBCO with an additional benzene ring. The study shows that introducing an additional benzene ring favors polymerization and disfavors depolymerization, and the effect is predominantly entropic. The benzo‐effect can be leveraged to fine‐tune the thermodynamics of polymerization and depolymerization to facilitate the chemical recycling of polymers.

## Graphical abstract

**The thermodynamics of polymerization** for *trans*‐benzocyclobutene‐fused cyclooctene is studied, and the enthalpy and entropy changes are compared to those for *trans*‐cyclobutane‐fused cyclooctene. It is found that while enthalpy is not significantly impacted by the presence of benzene ring, the entropy change at 1.0 M is flipped from negative to positive when the benzene ring is installed.

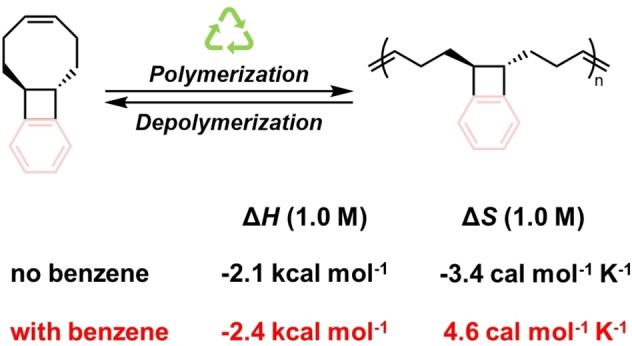

## Introduction

Synthetic polymers, such as plastics and synthetic rubbers, have become an indispensable part of modern life, because of their outstanding stability and mechanical properties. However, the chemical stability of synthetic polymers also makes them extremely durable, causing the accumulation of plastics in the environment.[^1^, ^2^, ^3^, ^4^] To address the environmental issues, it is desirable to recycle plastics after their use. Nevertheless, up to now, only a small fraction (∼9%) of polymer materials are recycled, mostly through mechanical recycling, which causes deterioration in the performance of materials and is not suitable for recycling of mixed materials such as composites.^[5]^ Chemical recycling the polymers into their constituents is a promising strategy, as it is suitable for mixed materials and allows for the regeneration of materials of quality comparable to that of virgin polymers. The circular plastic economy not only alleviates environmental pollution but also allows the limited resources to be efficiently used.[^6^, ^7^, ^8^]

A suitable chemical recycling system requires the depolymerization conditions to be efficient in energy use while side reactions are minimized. Meanwhile, the polymer needs to be stable under the conditions for storage and use. Balanced stability and recyclability require a judicious design of the system to achieve appropriate thermodynamics in polymerization and depolymerization. Most polymerizations feature favorable enthalpy change and unfavorable entropy change, i. e., Δ*H* <0 and Δ*S* <0. Therefore, there is a certain temperature, termed ceiling temperature (*T*_c_), at which the two factors canceled out.

Chemically recyclable polymers that are based on ring‐opening polymerization of cyclic monomers have received significant attention in the past few years.[^9^, ^10^, ^11^, ^12^, ^13^, ^14^, ^15^] Demonstrated systems include lactones,^[10]^ thiolactones,^[11]^ cyclic carbonate,^[12]^ cyclic acetals,^[13]^ and cyclic olefins.[^14^, ^15^] The polymerization thermodynamics of these systems can be tuned by editing the chemical structures of the cyclic monomers, including varying the size of the ring, adding substituents on the ring,^[11]^ and introducing fused rings.^[10]^ Cyclic olefins are desirable monomers for chemically recyclable polymers, as they allow for the preparation of polymers with hydrocarbon backbone, which have greater hydrolytic stability compared to other systems. The ruthenium‐based catalysts developed by Grubbs and others allow for ring‐opening metathesis polymerization (ROMP) and ring‐closing metathesis depolymerization to be conducted under mild or ambient conditions. However, the monomers for depolymerizable polyalkenamers were limited to five‐membered cyclic olefins; the low glass transition temperatures (*T*_g_s) of these polymers limit their applications.^[16]^ Moreover, since the thermodynamics for the polymerization of cyclopentene can be drastically affected by substituents, the preparation of many substituted polycyclopentenes require extremely low temperatures.^[17]^ Furthermore, living polymerization of cyclopentene has proved challenging, and existing demonstrations of controlled polymerization of cyclopentene either require the conversion to be limited to a low level^[18]^ or need to be done in a variable‐temperature fashion.^[19]^

**Figure 1:**
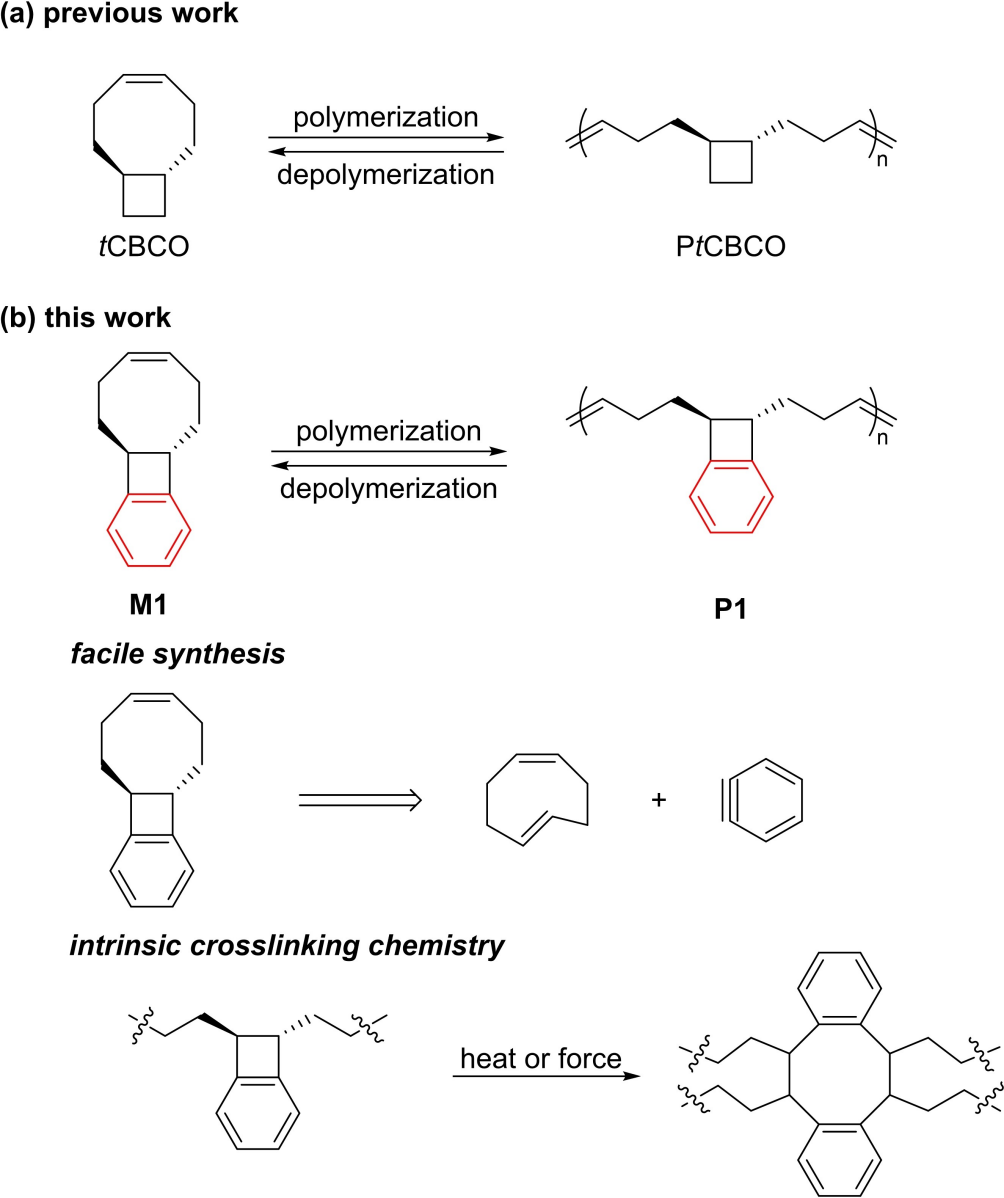
Chemically recyclable polymer systems based on (a) *trans*‐cyclobutane‐fused cyclooctene (*t*CBCO) and (b) *trans*‐benzocyclobutene‐fused cyclooctene (**M1**). **M1** can be conveniently synthesized from benzyne and 1,5‐*cis*, *trans*‐cyclooctadiene, and benzocyclobutene can be used as a crosslinker.

To address the challenges associated with the five‐membered cyclic olefins, we have recently demonstrated that introducing an appropriate fused ring (e. g., a *trans*‐cyclobutane, Figure 1a) to polycyclooctene can convert the otherwise nondepolymerizable polymer into a depolymerizable one.^[20]^ The calculated ring strain energy (RSE) of *trans*‐cyclobutane‐fused cyclooctene (*t*CBCO) is 4.9 kcal/mol, 3.3 kcal/mol lower than that of an unsubstituted cyclooctene (8.2 kcal/mol) and comparable to that of cyclopentene (5.2 kcal/mol).^[20]^ The *T*_g_ of the polymers can be adjusted from −30 °C to 100 °C by varying the functional groups on cyclobutane, allowing for both elastic and plastic polymeric materials to be prepared.^[20]^ In addition, *cis*‐cyclooctene can be isomerized into its *trans* analogue, which is suitable for living ROMP and allows for the preparation of depolymerizable polymers with diverse architectures, including block copolymers and graft polymers.[^21^, ^22^, ^23^]

A system relevant to *t*CBCO is *trans*‐benzocyclobutene‐fused cyclooctene (**M1**, Figure 1b), which can be viewed as adding a benzene ring next to cyclobutane in *t*CBCO. Compared to *t*CBCO, the synthesis of which requires a photochemical [2+2] cycloaddition of 1,5‐*cis*,*cis*‐cyclooctadiene with an olefin and often gives low yield (<40%),^[24]^ **M1** is advantageous in that its synthesis can be conveniently conducted thermally using benzyne and 1,5‐*cis*,*trans*‐cycloooctadiene at high yield.^[25]^ In addition, benzocyclobutene can undergo thermally or mechanically induced electrocyclic ring opening to form *ortho*‐quinodimithide, which is highly reactive and can be used for crosslinking.[^26^, ^27^, ^28^] Our previous studies show that introducing an additional ring – such as succinimide and cyclohexane – next to cyclobutane does not drastically affect the depolymerizability of the corresponding polymer.[^20^, ^29^] However, in those systems, the additional rings do not change the type of hybridization; introducing benzene ring is different, as it changes two sp^3^ carbons in cyclobutane into two sp^2^ carbons. Also, since benzene ring is more rigid than succinimide and cyclohexane, could it impact the thermodynamics of polymerization/depolymerization more profoundly? Motivated by these questions and the benefits of using benzocyclobutene, we studied the thermodynamics of polymerization of **M1** and compare it to that of *t*CBCO. We found that while introducing the benzene ring does not significantly impact Δ*H*, surprisingly, it flipped the Δ*S* at 1.0 M from negative to positive, rendering the depolymerization inaccessible at this concentration. Although introducing benzene ring negatively impacts depolymerization, this unique thermodynamic effect provides insight into designing monomers for chemically recyclable polymers.

## Results and Discussion

At first, we calculated the RSE of **M1** using a previously reported method,^[30]^ which gives a value of 5.2 kcal/mol (Table S1), comparable to that of *t*CBCO. The favorable RSE of **M1** further encouraged us to study the thermodynamics of polymerization and depolymerization.

**Figure 2:**
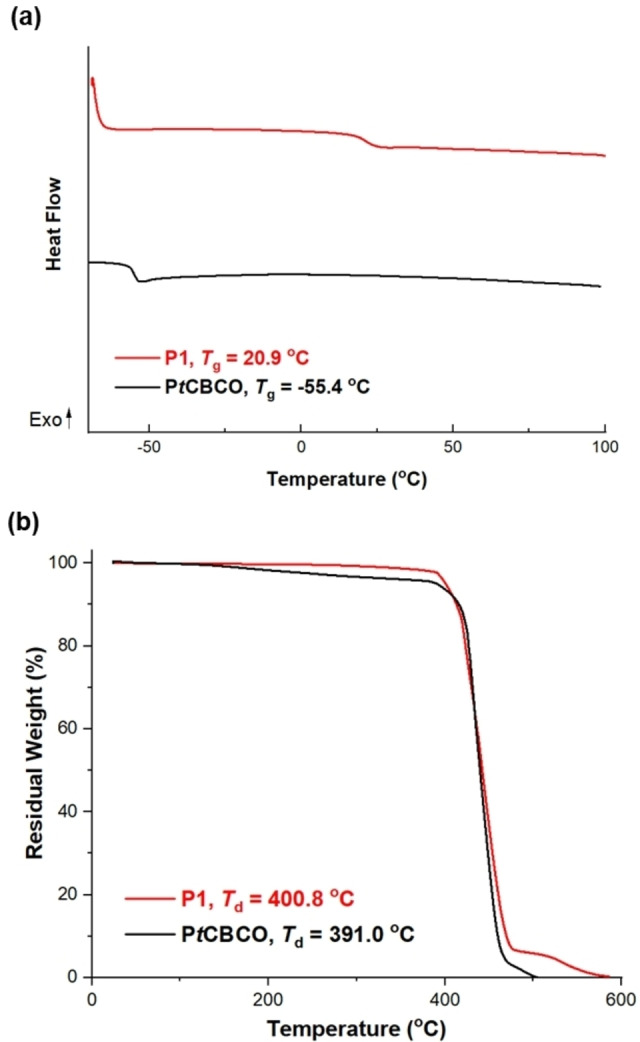
Thermal properties of **P1**. (a) TGA and (b) DSC traces of **P1**. The thermal properties of P*t*CBCO were reported in Ref. [32].

**M1** was synthesized according to a previously reported procedure.^[31]^ With the monomer in hands, we conducted a polymerization using Grubbs 2nd‐generation catalyst (**G2**) as the initiator. The polymerization was conducted in dichloromethane under ambient conditions, with an initial monomer concentration of 1.8 M and a monomer‐to‐initiator ratio of 600 : 1 or 1000 : 1. The polymerization was allowed to proceed for 3 h before it was quenched with ethyl vinyl ether and purified by precipitation in MeOH. The molecular weight information and thermal properties of the resulting polymer **P1** (Figure 1b) were collected and summarized in Table 1. Those of P*t*CBCO are also listed in Table 1 and for comparison.^[32]^ The *T*_g_ of **P1** is ∼75 °C higher than that of P*t*CBCO (Figure 2a) because of the additional benzene substitution. Similar to P*t*CBCO, **P1** has excellent thermal stability, with a decomposition onset temperature *T*_d_ of 401 °C (Figure 2b).

**Table 1:** Molecular Weight Information and Thermal Properties of **P1**.

Entry	*M*_n_ (kDa)^[b]^	*Đ* ^[c]^	*T*_d_ [°C]^[d]^	*T_g_* [°C]^[e]^
**P1**	57	1.60	401	21
P*t*CBCO^[a]^	58	1.61	391	−55

[a] The molecular weight information and thermal properties of P*t*CBCO were reported in Ref. [32]. [b] *M*_n_ is determined by gel permeation chromatography (GPC) in THF using standard molecular weight method. [c] *Ð*, dispersity, *M*_w_/*M*_n_. [d] *T*_d_, the decomposition onset temperature defined as the temperature at which the polymer experiences 5% weight loss, measured by thermogravimetric analysis (TGA). [e] *T*_g_, glass transition temperature, measured by differential scanning calorimetry (DSC).

**Figure 3:**
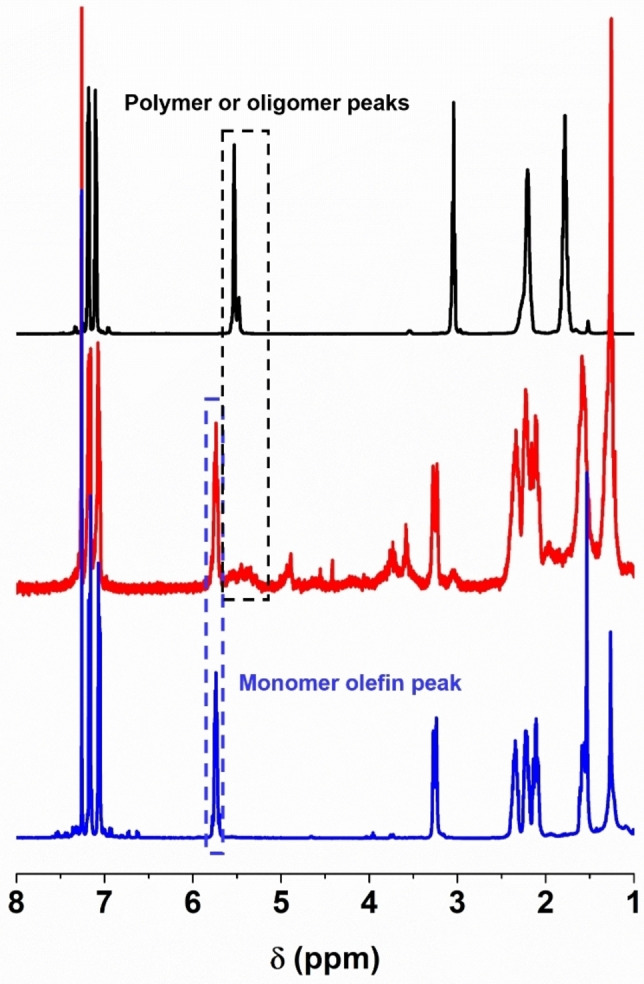
Depolymerization of **P1**. ^1^H NMR spectra for **P1** before (black) and after (red) the solution was heated at 50 °C for 2 h in the presence of 1 mol % **G2**. The spectrum of **M1** is shown in blue as a reference.

According to our previous study of *t*CBCO system, we firstly conducted the depolymerization in moderate concentration (∼200 mM) at 50 °C in CHCl_3_, we didn't observe monomer signal at 5.8∼5.7 ppm on ^1^H NMR. Monomer recovery (∼63.7%) was observed when the depolymerization was conducted at a substantially lower concentration (5 mM, Figure 3). The depolymerization conversion of P*t*CBCO reached 98% at this condition. Compared to P*t*CBCO, the reduced depolymerization efficiency motivated us to investigate the thermodynamics in the polymerization of **M1**.

To obtain the thermodynamics for the polymerization of **M1**, a 1.0 M solution of **M1** in xylenes was made, and 1.0 mol% **G2** was added, and the polymerization was monitored with ^1^H NMR. However, no residual monomer signal was observed on the ^1^H NMR, even when the solution was heated at 60 °C, suggesting a complete conversion of the monomer. To obtain an equilibrium between polymerization and depolymerization, we decreased the concentration of the monomer to 0.1 M. With this low concentration (∼1 wt%), it is necessary to remove solvent before NMR characterization. To facilitate solvent removal, we switched to dioxane, a solvent with relatively lower boiling point for the thermodynamic study. The polymerization of **M1** was conducted at five temperatures (18 °C, 35 °C, 45 °C, 55 °C and 65 °C), at an initial monomer concentration of 0.1 M in dioxane with 1 mol% of **G2**. The study was repeated three times at each temperature.

Before being quenched with ethyl vinyl ether, the polymerization was allowed to proceed for sufficient time for the system to reach an equilibrium. The concentration of the residual monomer at equilibrium ([M]_e_) was measured by ^1^H NMR, and the value at each temperature is listed in Table S2. The logarithm of [M]_e_ was plotted against the inverse of temperature (1/T), and the plot was fit to Eq. 1. The fit gave the thermodynamic parameters at 0.1 M: Δ*H*=−2.4±0.3 kcal mol^−1^, Δ*S*=0.04±0.81 cal mol^−1^K^−1^ (Table S3). Since concentration only impacts entropy of polymerization, the enthalpy change at 1.0 M remains identical to that at 0.1 M, and the entropy change at 1.0 M can be obtained by applying Eq. 2. The Δ*H* and Δ*S* for **M1** at 1.0 M are −2.4±0.3 kcal mol^−1^ and 4.6±0.8 cal mol^−1^ K^−1^ (Table 2), respectively. The proximity in Δ*H* values (−2.4 kal/mol vs. −2.1 kcal/mol) between **M1** and *t*CBCO is consistent with their comparable RSEs (5.2 kcal/mol vs. 4.9 kcal/mol). The lower Δ*H* value compared to RSE is likely caused by the formation of cyclic oligomers during the polymerization, which has been observed previously.^[20]^

(1) $$\ln{\left[M\right]}_{e}=\frac{\Delta H}{RT}-\frac{\Delta S}{R}$$

(2) $$\Delta S\;([M])=\Delta S\;\left({\left[M\right]}_{o}\right)-\mathrm{Rln}\frac{{\left[M\right]}_{o}}{\left[M\right]}$$

**Table 2:** Polymerization thermodynamic data for **M1** and *t*CBCO.

Entry	Δ*H* [kcal mol^−1^]	Δ*S* [cal mol^−1^ K^−1^]	*T*_c_ @ 1.0 M [°C]
**M1**	−2.4±0.3	4.6±0.8	–
*t*CBCO^[a]^	−2.1±0.1	−3.4±0.3	335

[a] Polymerization thermodynamic data of *t*CBCO were reported in Ref. [32].

The positive Δ*S* value (4.6±0.8 cal mol^−1^ K^−1^) at 1.0 M means that the polymerization of **M1** is favorable entropically, unlike other cyclooctene monomers. Because of the positive Δ*S*, *T*_c_ does not exist at 1.0 M concentration. When the system is diluted to from 200 mM, Δ*S* is still positive (1.4 cal mol^−1^ K^−1^); when the concentration is further diluted 5 mM, Δ*S* is flipped to a negative value – −5.9 cal mol^−1^ K^−1^. The difference in Δ*S* values explains why the depolymerization was observed at 5 mM but not at 200 mM. The concentration dependence of ΔS also causes a concentration dependence of molecular weight. For example, polymerizations of M1 at concentrations of 0.3 M and 1.0 M gave polymers with molecular weights of 42 kDa and 143 kDa, respectively (Figure S10).

The Δ*S* comprises translational entropy change (Δ*S*_t_) and rotational entropy change (Δ*S*_r_). During polymerization, as monomers are connected to form a polymer chain, translational freedom is lost (Δ*S*_t_ <0). Δ*S*_t_ is sensitive to the concentration of monomer. As the concentration increases, the monomer becomes less mobile, and the loss in translational freedom is reduced (i. e., a lower value of Δ*S*_t_). At the same concentration, Δ*S*_t_ can be impacted by the size of the monomer. Since **M1** is expected to have similar size as *t*CBCO‐based monomers, the difference in their entropy should be caused by the difference Δ*S*_r_. In a ring‐opening polymerization, as the cyclic monomer is converted into a linear polymer, the system gains rotational freedom: Δ*S*_r_ >0. The more positive Δ*S*_r_ of **M1** than *t*CBCO means that more rotational freedom is gained during polymerization, which can be ascribed to the more rigid structure of the benzocyclobutene system.

## Conclusion

Establishing the design principle is critical in developing next‐generation chemically recyclable polymers. In this regard, the investigation of *trans*‐benzocyclobutene‐fused cyclooctene as a potential monomer for chemically recyclable polymer helps to understand the effect of introducing benzene fused ring on the thermodynamics of polymerization. Introduction of the benzene ring favors polymerization and disfavors depolymerization as it increases the magnitude of rotational freedom that is released during polymerization. The benzo‐effect complements the previously demonstrated remote *gem*‐disubstituent effect,^[32]^ which facilitates depolymerization, and therefore enriches the toolkit for tuning the performance of chemical recycling.

## Experimental Section

All essential experimental procedures and data can be found in the Supporting Information.

## Conflict of interest

The authors declare no conflict of interest.

1

## Supporting information

As a service to our authors and readers, this journal provides supporting information supplied by the authors. Such materials are peer reviewed and may be re‐organized for online delivery, but are not copy‐edited or typeset. Technical support issues arising from supporting information (other than missing files) should be addressed to the authors.

Supporting Information

## Data Availability

The data that support the findings of this study are available in the supplementary material of this article.
