# Supplementary material for: Evaluating Trans‐Benzocyclobutene‐Fused Cyclooctene as a Monomer for Chemically Recyclable Polymer
Source: Chem Asian J. 2023 Jan 3;18(3):e202201133. doi: 10.1002/asia.202201133 (PMC10107132; doi:10.1002/asia.202201133)
Supplement: Supplementary file 1 — Supporting Information [file ASIA-18-0-s001.pdf]

CHEMISTRY
AN ASIAN JOURNAL

Supporting Information

Evaluating *Trans*-Benzocyclobutene-Fused Cyclooctene as a Monomer for Chemically Recyclable Polymer

Hsin-Wei Su+, Junfeng Zhou+, Seiyoung Yoon, and Junpeng Wang*This manuscript is part of a special collection on Sustainable Solutions for Plastic Issues.© 2022 The Authors. Chemistry - An Asian Journal published by Wiley-VCH GmbH. This is an open access article under the terms of the Creative Commons Attribution License, which permits use, distribution and reproduction in any medium, provided the original work is properly cited.

Table of Contents

Materials and instruments....3
Synthesis....3
Ring strain energies of the monomers....3
Polymerization thermodynamic studies....4
Depolymerization studies....6
DSC, TGA and GPC traces....8
Optimized geometries....10
Reference....12

Materials and instruments

Hydrogen peroxide, trifluoromethanesulfonic anhydride, sodium hydride, Grubbs 2nd-generation catalyst (M204), *n*-butyllithium (2.5 M in hexane) and ethyl vinyl ether were purchased from Sigma Aldrich. Triphenylphosphine, cesium fluoride, 2-bomophenol, 1,1,1,3,3,3-hexamethyldisilazane and trifluoromethanesulfonic anhydride were purchased from TCI. *tert*-Butyl chloride and lithium wire were purchased from Alfa Aesar. $CDCl_3$ were purchased from Cambridge Isotope Laboratories. Silicycle F60 (230-400 mesh) silica gel was used for flash column chromatography. All solvents and other chemicals were purchased from Fisher Chemical and used without further purification unless specified.

^1H NMR spectra were collected on a Varian 500 MHz spectrometer by using $CDCl_3$ as the solvent. Gel permeation chromatography (GPC) analyses were carried out on the Tosoh EcoSEC HLC-8320 GPC using preservatives free HPLC grade THF (Fisher Chemical) as the eluent, with a flow rate of 1 mL min^{-1} at 40°C. The system was calibrated using polystyrene standards (PStQuick, Tosoh). Differential scanning calorimetry (DSC) was determined with a TA Instrument of DSC-TA Discovery DSC 250 at a heating rate of 10 °C/min under nitrogen flow. Thermogravimetric analysis (TGA) was performed on a TGA -TA Discovery TGA 550 apparatus with a heating rate of 20 °C/min under nitrogen atmosphere.

Synthesis

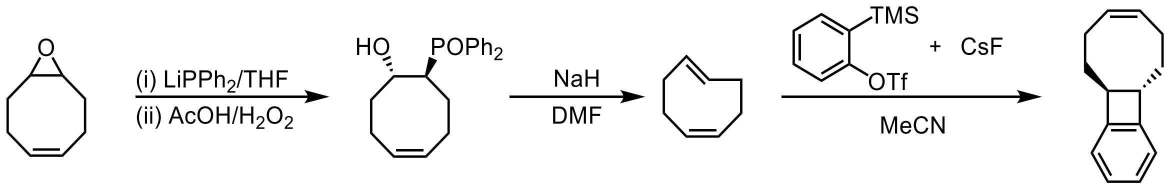

M1

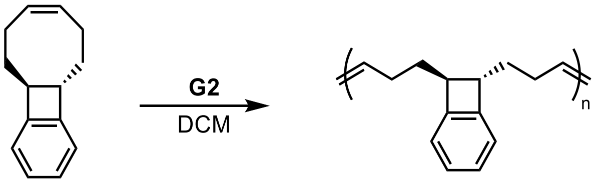

M1 **P1**

Benzicyclobutane fused cyclooctene **M1** and the corresponding polymer **P1** were prepared according to a reported procedure.[1,2]

Ring strain energies of the monomers

The ring strain energies of the monomers were calculated based on a previously reported method.[3-5]

Table S1. The calculated ring strain energies (RSEs) for *t*CBCO and **M1** used in this work.

Entry	Monomer structures	RSE (kcal mol^{-1})

*t*CBCO	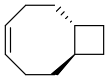	4.9
**M1**	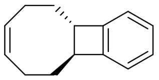	5.2

Polymerization thermodynamic studies

To a 1-dram vial was added **G2** (4.3 mg, 0.005 mmol, 0.01 equiv.) and 5.08 mL dioxane to make a catalyst stock solution. **M1** (93.7 mg, 0.508 mmol, 1.0 equiv.) was weighed in another 1-dram vial. The catalyst solution was transferred into the vial to prepare a 0.1M polymerization solution. The mixture was stirred for 3 min, and the resulting solution was divided into 5 vials, with each vial containing ~1.0 mL reaction solution. These five vials were placed in five *pre*-heated oil baths at different temperatures, ranging from room temperature (18 °C, measured by a thermometer), 35 °C, 45 °C, 55 °C and 65 °C. The above procedure was repeated twice so that three parallel samples were studied at each temperature. Kinetic study for the polymerization of **M1** was performed to obtain the time required for the system to reach equilibrium. Each polymerization for the thermodynamic studies was allowed to proceed sufficient time before being quenched with 30 μL ethyl vinyl ether. The resulting mixture was concentrated on rotavapor and re-dissolved in $CDCl_3$ for NMR analysis. The monomer concentration at the equilibrium was determined by ^{1}H NMR.

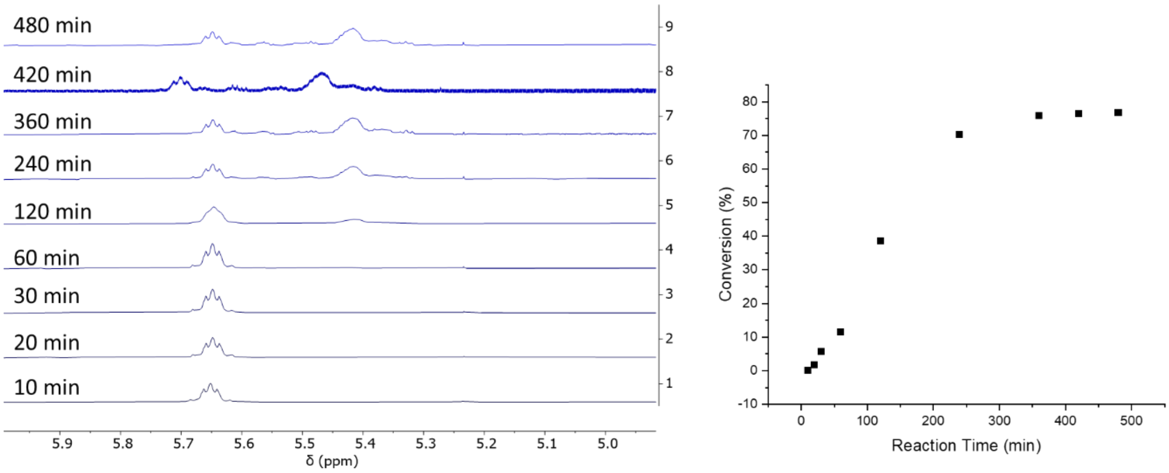

Figure S1. Kinetic study for the polymerization of **M1** at 25 °C: (left) stacked partial ^{1}H NMR spectra; (right) plot of conversion against reaction time.

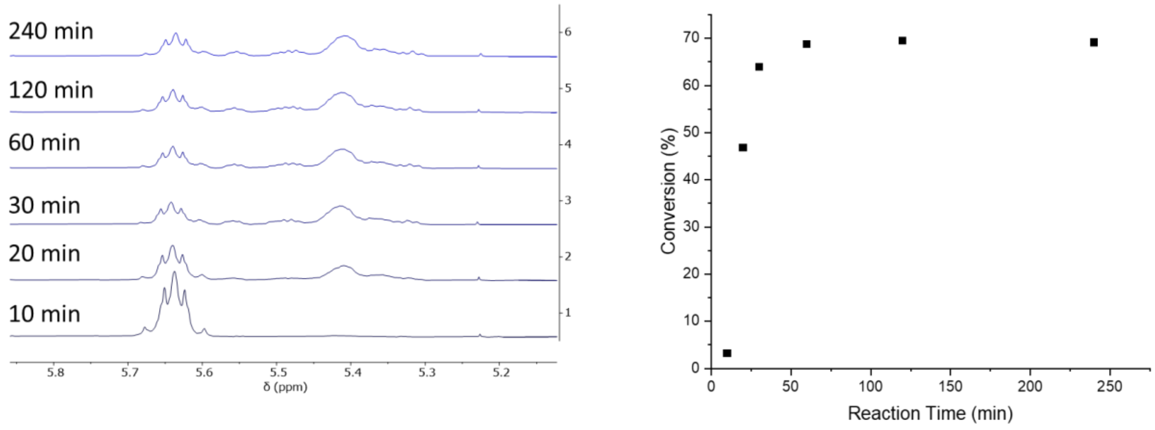

Figure S2. Kinetic study for the polymerization of **M1** at 35 °C: (left) stacked partial 1H NMR spectra; (right) plot of conversion against reaction time.

The thermodynamic parameters can be extracted by the linear fitting of the plot of $\ln[M]_e$ against $1/T$ according to the following equation:

$$\ln[M]_e = \frac{\Delta H}{RT} - \frac{\Delta S}{R}$$

Here $[M]_e$ is the monomer concentration at equilibrium in mol L^{-1}, T is the reaction temperature in K, ΔH is the enthalpy change of polymerization in kcal mol^{-1}, ΔS is the entropy change of polymerization in cal mol^{-1} K^{-1}, and R is the gas constant (1.987 cal mol^{-1} K^{-1}).

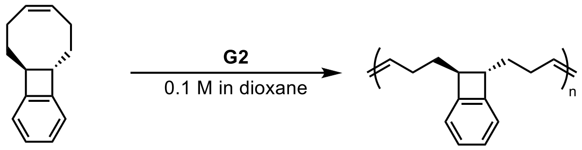

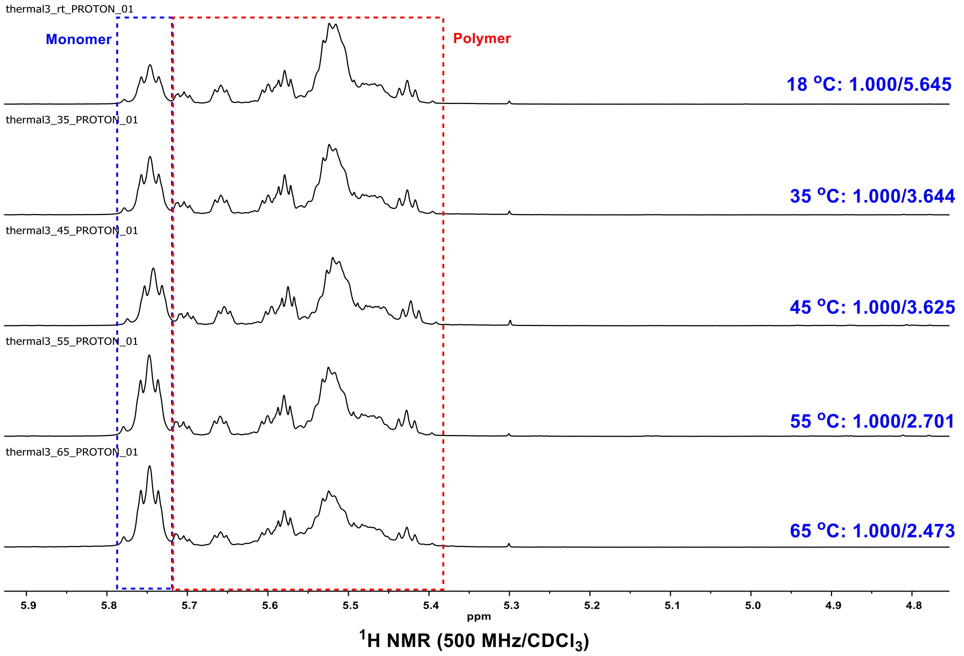

Figure S3. Partial ^1H NMR spectra (500 MHz, $CDCl_3$) for one of three parallel thermodynamic studies of **M1**.

Table S2. Average of monomer concentration at equilibrium $[M]_e$ (mol/L) for **M1** at Different Temperatures

**Temperature**	**RT (18°C)**	**35°C**	**45°C**	**55°C**	**65°C**
**M1**	0.016±0.001	0.020±0.002	0.022±0.001	0.027±0.000	0.027±0.002

Table S3. Polymerization thermodynamic data for **M1**.

Concentration	Δ*H* (kcal mol^{-1})[a]	Δ*S* (cal mol^{-1} K^{-1})	T_c (°C)
0.1 M	-2.39±0.25	0.04±0.81	--
1.0 M	-2.39±0.25	4.61±0.81	--
0.005 M	-2.39±0.25	-5.92±0.81	130.5

[a.] Assuming enthalpy change is not affected by concentration

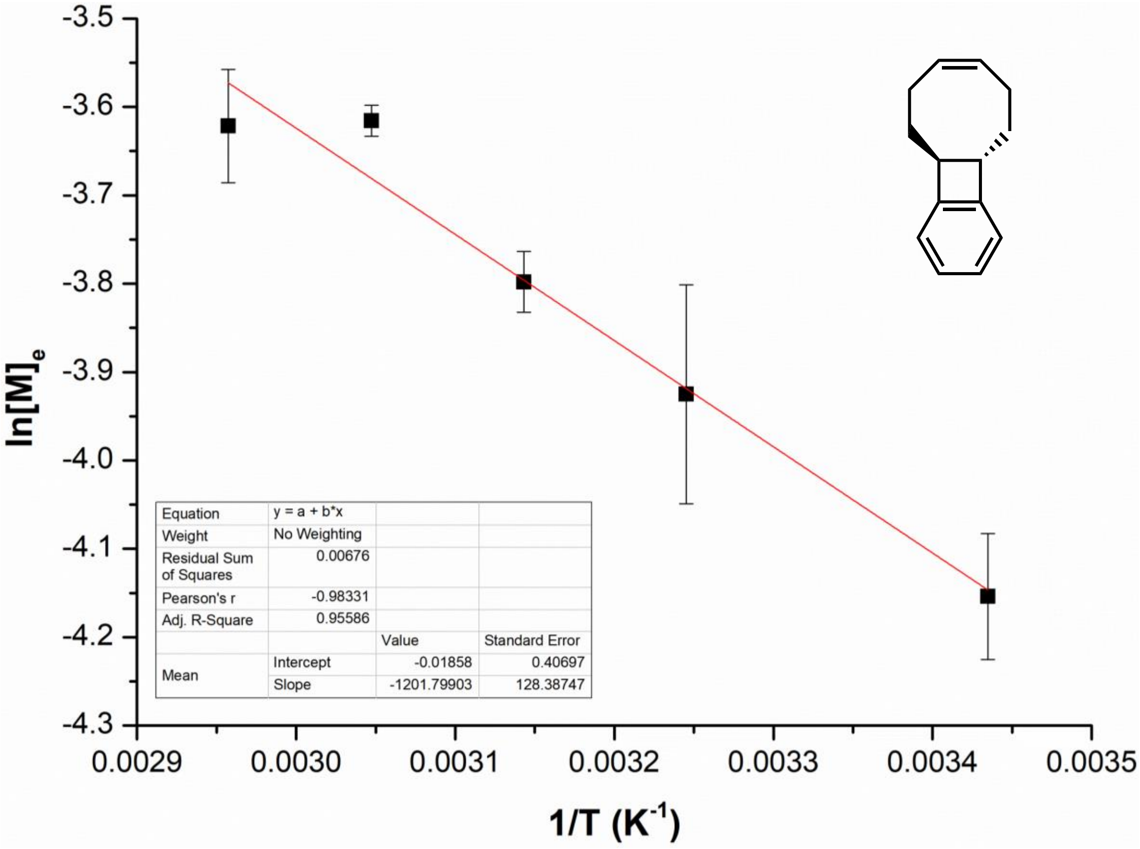

Figure S4. The van ‘t Hoff plot of **M1**.

Depolymerization studies

According to the previous kinetic study,[4,5] 30 min is enough for depolymerization reaching the equilibrium at 50 °C in the presence of 1 mol% **G2**. To ensure the depolymerization to reach equilibrium, the depolymerization for each polymer was allowed to proceed for 2 h at 50 °C in the presence of 1 mol% **G2**. The depolymerizations of **P1** were studied in $CDCl_3$ at [olefin] = 5 mM at 50 °C and room temperature. A representative procedure of depolymerization is described as follows.

P1 (8.0 mg, 43.41 μmol, 1.00 equiv. based on the repeat unit) was dissolved in 8.0 mL $CHCl_3$ in a 1-dram vial equipped with a stir bar. **G2** (0.37 mg, 0.43 μmol, 0.01 equiv.) in 0.68 mL $CHCl_3$ was

added to the solution of **P1**, resulting in a 5 mM olefin concentration with 1 mol/% of **G2**. The vial was placed in a *pre*-heated oil bath at 50 °C and kept at this temperature for 2 h. Each reaction was quenched with $CHCl_3$ solution of ethyl vinyl ether, and the resulting mixture was concentrated, dried, re-dissolved in $CDCl_3$, and characterized by ^1H NMR. The conversion was determined from the integrations of the olefinic peaks for monomers and polymer/oligomers.

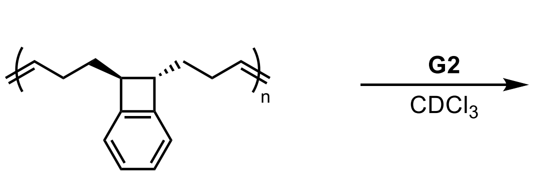

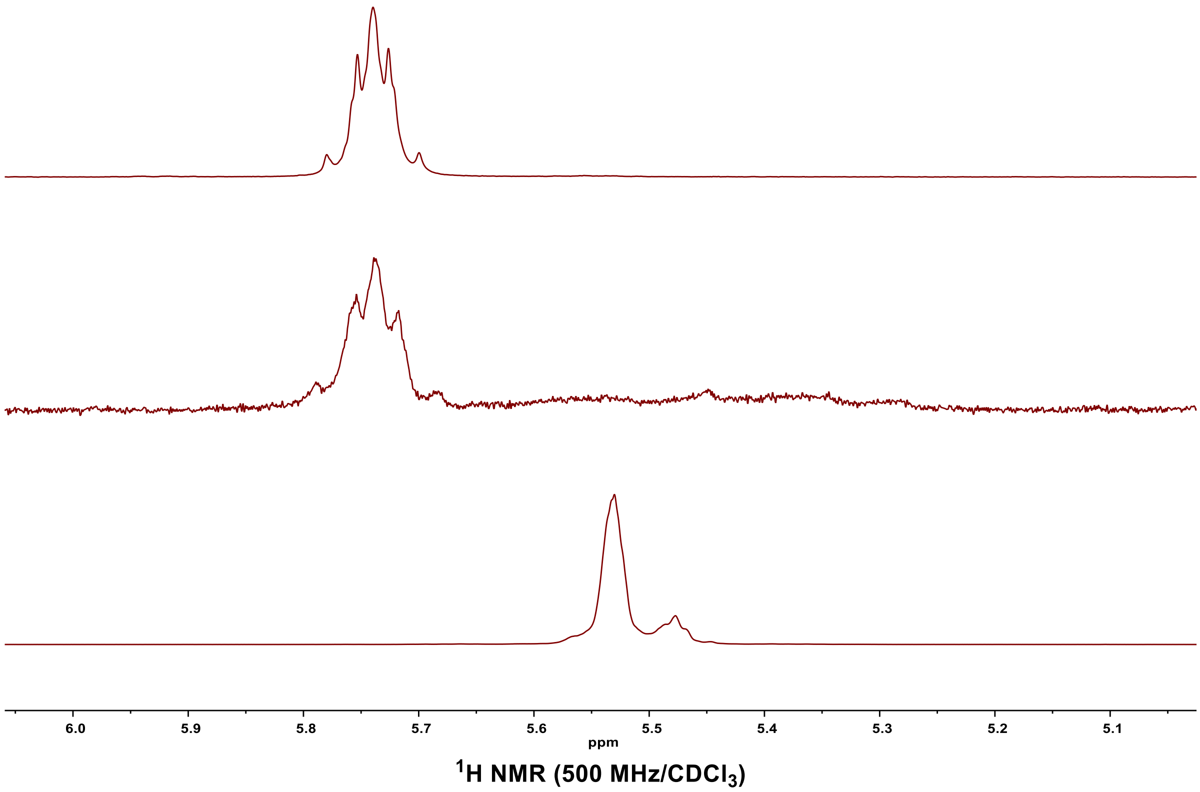

Figure S5. Partial ^1H NMR spectra (500 MHz, $CDCl_3$) for depolymerization studies at [olefin] = 5 mM of **P1**. The ^1H NMR spectra of monomer (top) and polymer (bottom) are included as references.

DSC, TGA and GPC traces

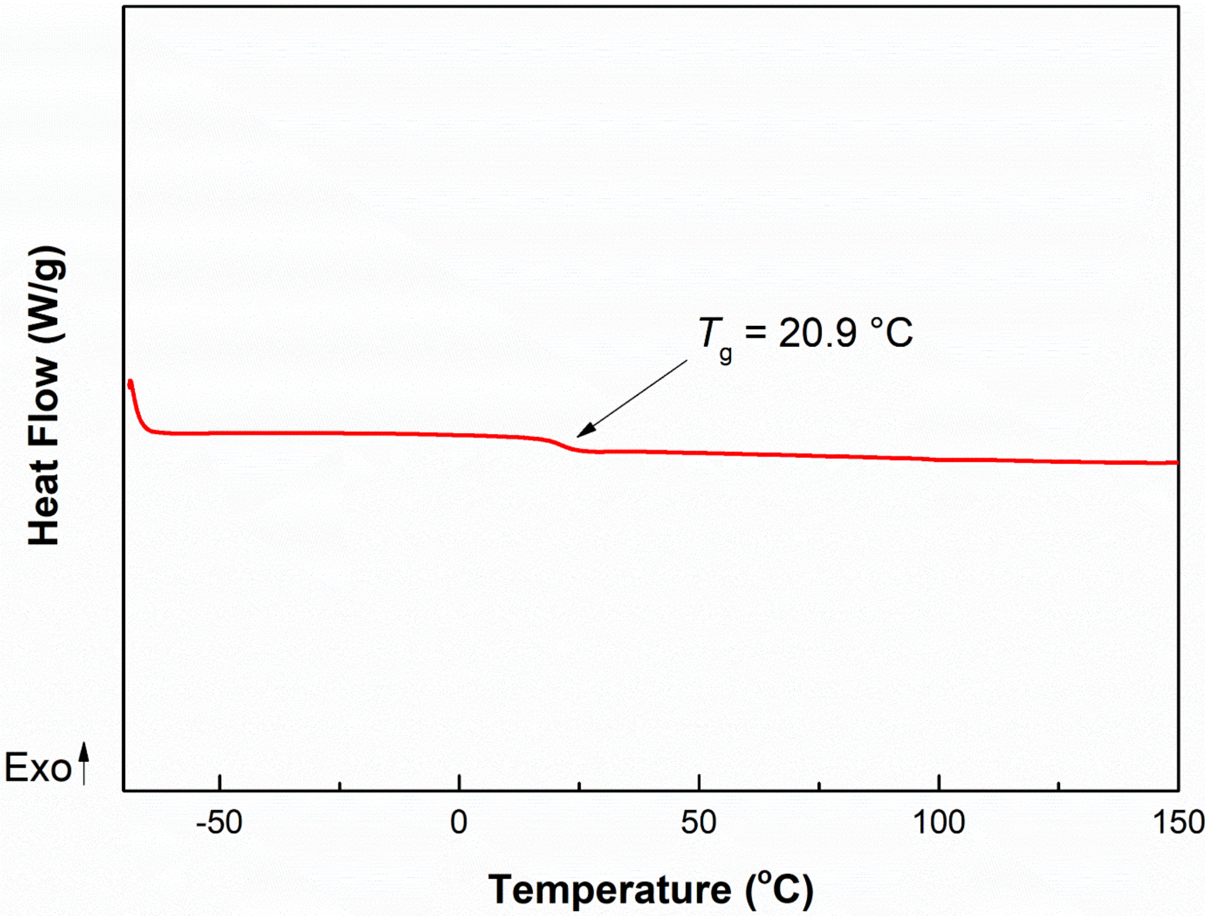

Figure S6. DSC curve of **P1**.

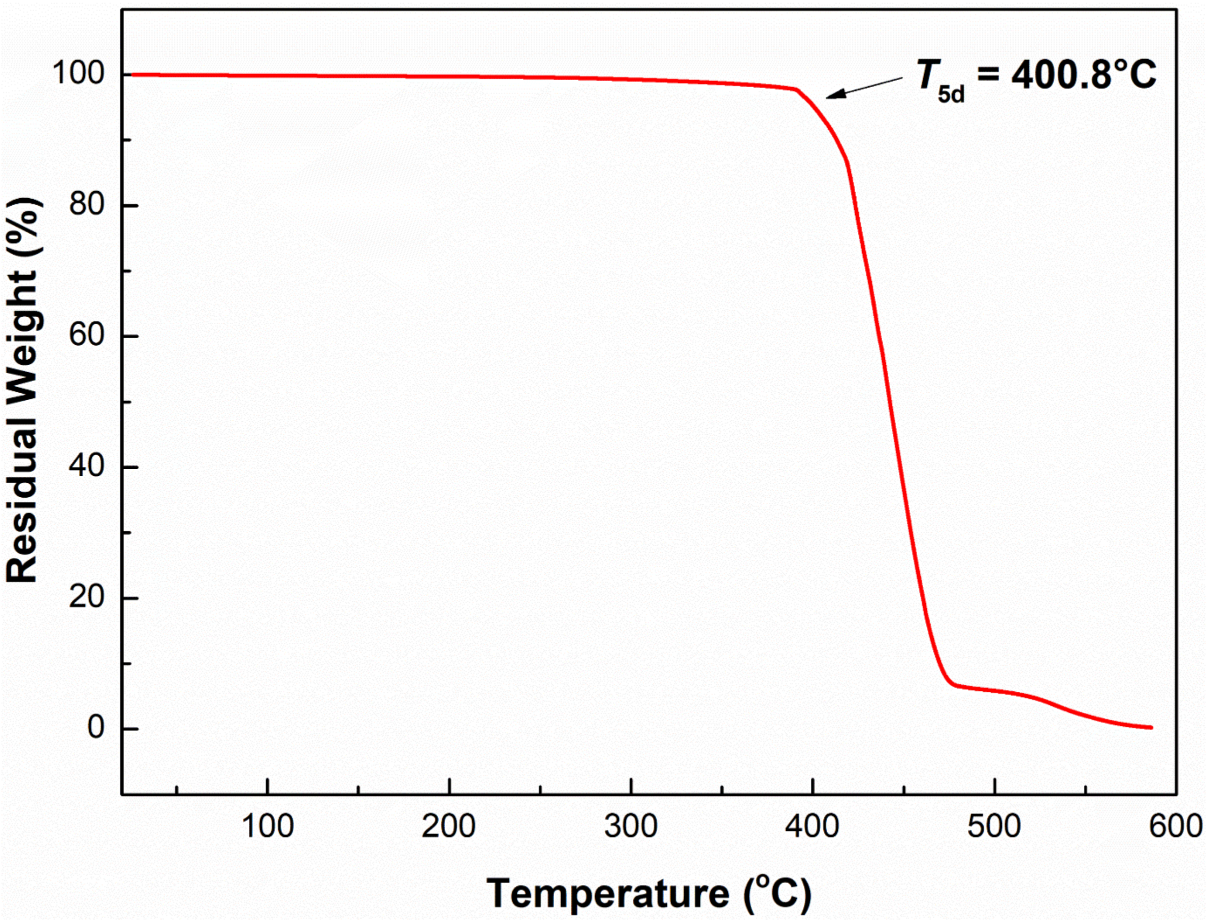

Figure S7. TGA curve of **P1**.

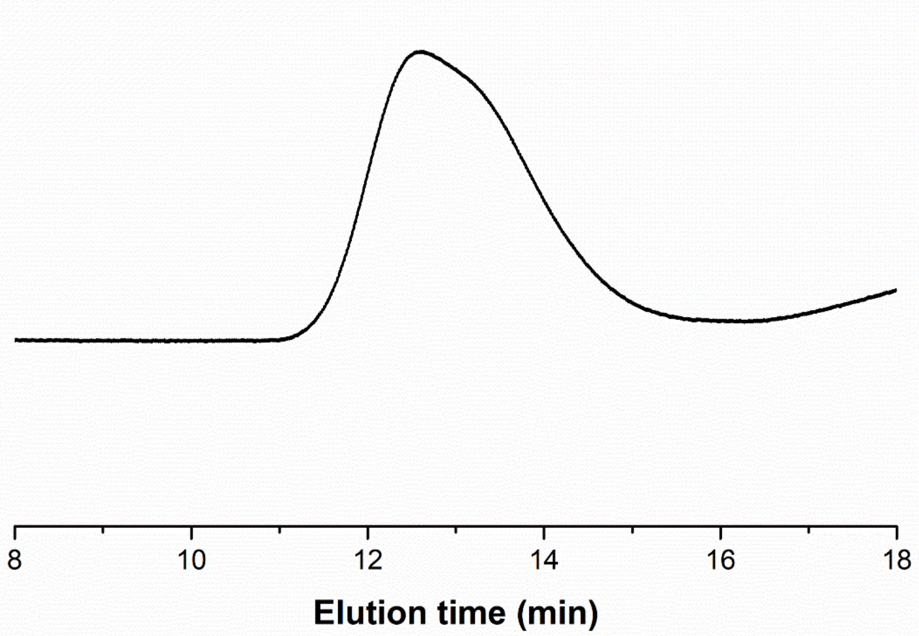

Figure S8. GPC trace of **P1**.

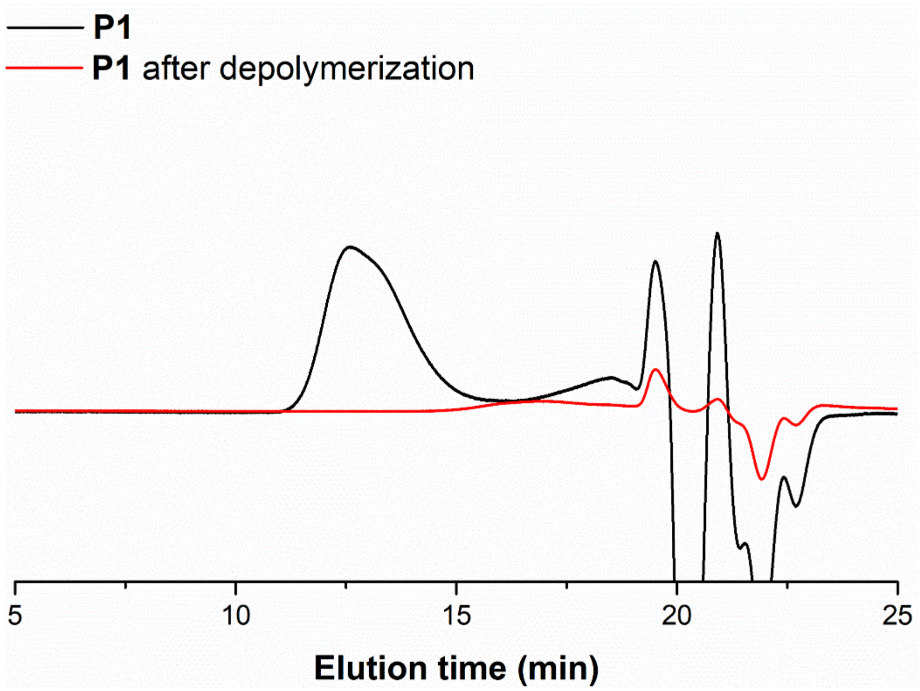

Figure S9. GPC traces for polymers before (black) and after (red) depolymerization ([olefin] = 5 mM).

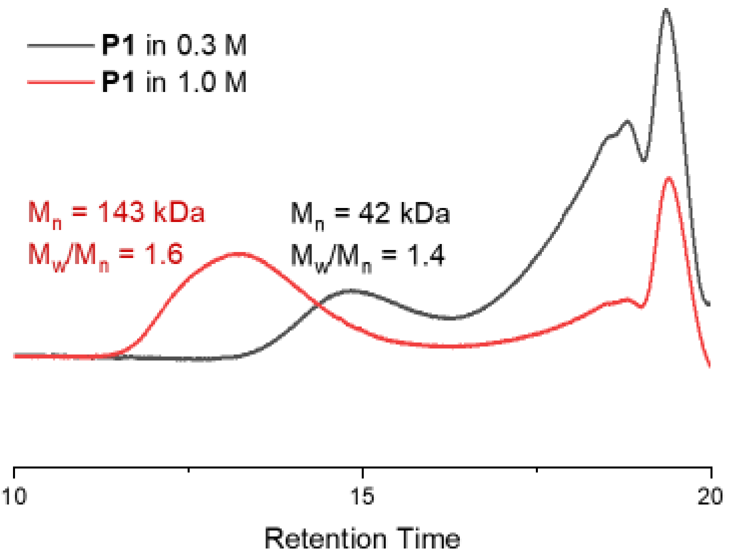

Figure S10. GPC traces for polymers made from initial monomer concentrations of 0.3 M (in black) and 1.0 M (in red). Monomer-to-initiator ratio was set at 600 for both polymerizations.

Optimized geometries

Optimized geometries from DFT calculations at B3LYP/6-31g(d,p) level.

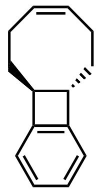

C	-0.1714890000	0.7176940000	0.4418640000
C	1.3418880000	0.6471370000	0.2568140000
C	1.3096760000	-0.6630090000	-0.2246770000
C	-0.2138880000	-0.7204150000	-0.2291210000
C	-0.9995800000	1.8296890000	-0.2042010000
C	-0.9908900000	-1.8166760000	0.4996390000
C	-2.5239640000	1.6517830000	0.0192870000
C	-3.1611030000	0.5072040000	-0.7423710000
C	-2.4524420000	-1.3812700000	0.7944010000
C	-3.1522740000	-0.7891660000	-0.4033540000
H	-0.4333940000	0.6544600000	1.5079460000
C	2.4654370000	-1.3639650000	-0.5387720000
C	3.6749360000	-0.6726600000	-0.3590430000
C	3.7058260000	0.6490770000	0.1064410000
C	2.5289130000	1.3449010000	0.4285540000
H	-0.6018800000	-0.6333900000	-1.2503130000
H	-0.6974010000	2.7962220000	0.2207480000
H	-0.7806640000	1.8795650000	-1.2785040000
H	-0.9859760000	-2.7324640000	-0.1057940000
H	-0.5002520000	-2.0656110000	1.4489540000
H	-3.0269550000	2.5826240000	-0.2661370000
H	-2.6974550000	1.5311930000	1.0970830000
H	-3.6627940000	0.7819370000	-1.6698350000
H	-3.0129300000	-2.2464420000	1.1679230000
H	-3.6411390000	-1.4952560000	-1.0744840000
H	2.4590100000	-2.3854140000	-0.9096390000
H	4.6130050000	-1.1704160000	-0.5896790000
H	4.6667390000	1.1427650000	0.2237660000
H	2.5702590000	2.3668700000	0.7958960000
H	-2.4344490000	-0.6485880000	1.6093040000

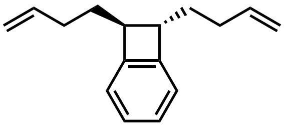

C	-0.4928450000	-1.0939580000	-0.0998460000
C	0.9843470000	-1.4719510000	-0.1150720000
C	1.4193350000	-0.1515890000	-0.0031410000
C	-0.0002750000	0.4140450000	0.0304170000
C	-1.3411280000	-1.6523960000	1.0455390000

C	-0.4184100000	1.3633540000	-1.0957620000
C	-2.7779410000	-1.0915980000	1.0994000000
C	-3.6213240000	-1.4754600000	-0.0858760000
C	0.1993540000	2.7739260000	-0.9954010000
C	-0.2681170000	3.5519590000	0.2046950000
H	-0.9999660000	-1.2718740000	-1.0569920000
C	2.7673550000	0.1792950000	0.0413860000
C	3.6710810000	-0.8932590000	-0.0328450000
C	3.2318520000	-2.2197940000	-0.1411250000
C	1.8656620000	-2.5419620000	-0.1827550000
H	-0.2642090000	0.8521060000	1.0017220000
H	-1.3859410000	-2.7467660000	0.9557670000
H	-0.8385990000	-1.4448990000	1.9986710000
H	-0.1376000000	0.9210180000	-2.0598130000
H	-1.5139660000	1.4465700000	-1.1007950000
H	-2.7502950000	0.0008570000	1.1955660000
H	-3.2555640000	-1.4719370000	2.0138550000
H	-3.7157900000	-2.5473820000	-0.2700350000
H	1.2935460000	2.7003360000	-0.9884170000
H	-1.3455980000	3.7156140000	0.2697620000
H	3.1270300000	1.2000750000	0.1370880000
H	4.7388720000	-0.6939470000	-0.0015510000
H	3.9700400000	-3.0155640000	-0.1931480000
H	1.5405000000	-3.5756240000	-0.2659160000
H	-0.0691380000	3.3283660000	-1.9060500000
C	-4.2454680000	-0.6254350000	-0.9020090000
H	-4.8469030000	-0.9704730000	-1.7377780000
H	-4.1842050000	0.4509840000	-0.7580710000
C	0.5152180000	4.0388190000	1.1675300000
H	0.1116330000	4.5969760000	2.0071640000
H	1.5939720000	3.9016010000	1.1481670000

Reference

[1] Leitich, J. [2+2] cycloaddition of benzyne to *cis*, *trans*-1,5-cyclooctadiene and *cis*, *cis*-1,5-cyclooctadiene. *Tetrahedron Lett.* **1980**, *21*, 3025-3028.
[2] Wang, J.; Kouznetsova, T. B.; Niu, Z.; Ong, M. T.; Klukovich, H. M.; Rheingold, A. L.; Martinez, T. J.; Craig, S. L. Inducing and Quantifying Forbidden Reactivity with Single Molecule Polymer Mechanochemistry. *Nat. Chem.* **2015**, *7*, 323-327.
[3] Hejl, A.; Scherman, O. A.; Grubbs, R. H. Ring-opening metathesis polymerization of functionalized low-strain monomers with ruthenium-based catalysts. *Macromolecules* **2005**, *38*, 7214-7218.
[4] Sathe, D.; Zhou, J.; Chen, H.; Su, H.-W.; Xie, W.; Hsu, T.-G.; Schrage, B. R.; Smith, T.; Ziegler, C. J.; Wang, J. Olefin metathesis–based chemically recyclable polymers enabled by fused-ring

monomers. *Nat. Chem.* **2021**, *13*, 743-750.
[5] Zhou, J.; Sathe, D.; Wang, J. Understanding the Structure−Polymerization Thermodynamics Relationships of Fused-Ring Cyclooctenes for Developing Chemically Recyclable Polymers. *J. Am. Chem. Soc.* **2022**, *144*, 928-934.
